# Supplementary material for: Complexome profiling of the Chlamydomonas psb28 mutant reveals TEF5 as an early PSII assembly factor
Source: Plant Cell. 2025 Mar 17;37(6):koaf055. doi: 10.1093/plcell/koaf055 (PMC12164590; doi:10.1093/plcell/koaf055)
Supplement: koaf055_Supplementary_Data [file koaf055_supplementary_data.zip › psb28_interactive.zip!/plots/intensity/qualityPlots/peptides/ALD7_Cre13.g605650.t1.2.html]

### Cre13.g605650.t1.2

Trivial name: ALD7  
SeqCoverage [median]: 11.30  
Mass: 53.07 kDa  
  
 

| Sequence | MissedCleavage | Mass [Da] | wtIntensity | muIntensity | Score |
| --- | --- | --- | --- | --- | --- |
| GGSLPVINPATEKEFAR | 1 | 1784.94 | 0.00 | 836.36 | 38.61 |
| LVAQAAAANIRPASMELGGK | 1 | 1967.06 | 1132.13 | 2799.65 | 70.33 |
| VAFTGSAATGR | 0 | 1036.53 | 1416.45 | 1486.86 | 89.05 |
| VLSYVEAGK | 0 | 964.52 | 0.00 | 282.56 | 60.44 |
|  |  |  | 2548.6 | 5405.4 |  |
